# Study on the link between neutrophil percentage to albumin ratio and acute kidney injury in severe ischemic stroke patients during hospitalization

**DOI:** 10.3389/fneur.2025.1580248

**Published:** 2025-09-16

**Authors:** Chao Dong, Zhaobing Tang, Xingang Bai, Fangxu Que, Ling Bai, Yi Huang, Sheng He, Rizhao Pang

**Affiliations:** Department of Rehabilitation Medicine, The General Hospital of Western Theater Command, Chengdu, China

**Keywords:** NPAR, ischemic stroke, acute kidney injury, the EICU clinical research database, hospitalization

## Abstract

**Background:**

This study aimed to investigate the association between the neutrophil percentage-to-albumin ratio (NPAR) and the occurrence of acute kidney injury (AKI) in patients with severe ischemic stroke.

**Methods:**

Based on the EICU Clinical Research Database (EICU-CRD), 1,027 patients with severe ischemic stroke were enrolled (AKI group: 137 cases, non-AKI group: 890 cases). Data description: Non-normally distributed variables were expressed as median (IQR), and categorical variables were presented as frequency (weighted percentage). Statistical analysis: Intergroup comparisons were performed using the Wilcoxon rank-sum test and Rao-Scott chi-square test. Multivariate logistic regression and trend analysis were employed to evaluate the predictive value of NPAR for AKI, with adjustments for confounding factors.

**Results:**

1. NPAR levels: The AKI group exhibited significantly higher NPAR than the control group (29 ± 10 vs. 24 ± 7, *p* < 0.001). 2. Risk prediction: After adjusting for confounding factors including liver function, electrolyte levels, blood cell count, history of renal insufficiency, furosemide use, and vital signs, NPAR remained an independent risk factor for AKI (OR = 1.041, 95% CI: 1.007–1.076, *p*-value = 0.0162). 3. Dose-effect relationship: A significant increase in AKI risk was observed with each one-quarter increase in NPAR (Q4 vs Q1, OR: 3.598, 95% CI: 1.482–9.12, *p*-value: 0.0056, *p* for trend: 0.0028). 4. Subgroup analysis: The impact of elevated NPAR on AKI risk was more pronounced in male patients.

**Conclusion:**

Elevated NPAR levels significantly increase the risk of acute kidney injury in patients with severe ischemic stroke, demonstrating a clear dose–response relationship. These findings suggest that NPAR may serve as a potential biomarker.

## 1 Introduction

Ischemic stroke is brain tissue damage due to insufficient blood supply, with high incidence and mortality rates globally; statistics indicate that IS accounts for approximately 87% of all strokes. Recent studies have shown that IS patients often experience acute kidney injury (AKI), with patients having hypertension and chronic kidney disease at a higher risk of developing AKI after a stroke (1). Additionally, the occurrence of AKI is closely related to the inflammatory response following a stroke, as systemic inflammation triggered by a stroke may further exacerbate kidney damage (2). Epidemiological studies indicate that the incidence of AKI in IS patients can exceed 30%. A retrospective study found that approximately 30.18% of stroke patients in the intensive care unit (ICU) had AKI (3). Furthermore, another prospective observational study indicated that the incidence of AKI in acute IS was 34%, with rates as high as 66.7% in patients experiencing visceral bleeding (4). This suggests that monitoring and preventing AKI should be a high priority in the clinical management of stroke patients.

Research has shown that the activation and infiltration of peripheral immune cells after IS can exacerbate inflammation in the brain, particularly the activation of immune cells in the spleen significantly affects the level of inflammation in the brain (5). The systemic immune-inflammation index (SIII) is significantly correlated with the severity of IS, suggesting that systemic inflammatory responses may worsen brain injury (6). The release of inflammatory mediators such as cytokines and chemokines after a stroke may promote neuronal death and damage to the blood–brain barrier by activating endogenous inflammatory pathways (7). High-sensitivity C-reactive protein (CRP) is an acute-phase reactant whose levels significantly increase during inflammation, infection, and tissue injury, and it has been widely used to assess the inflammatory state in cardiovascular diseases and other chronic conditions (8). Elevated CRP levels are closely related to the risk of cardiovascular events and can serve as indicators for disease monitoring and prognosis assessment (9). Multiple studies have also shown that elevated inflammatory markers are associated with poor functional recovery after a stroke. Research has found that higher levels of the neutrophil-to-lymphocyte ratio (NLR) are closely related to poor functional prognosis in stroke patients, especially among those undergoing endovascular treatment (10). Inflammatory responses play a key role in the occurrence and development of acute kidney injury. In the pathophysiological mechanisms of AKI, inflammatory cell infiltration and the release of inflammatory factors are key pathological processes. Studies have shown that renal tubular cells release various inflammatory factors after injury, attracting immune cells (such as macrophages and neutrophils) to the damaged area, thereby aggravating the kidney injury (11).

However, the aforementioned inflammatory markers have certain limitations. First, most of these indicators discuss the relationship between inflammation and IS or AKI separately, without directly addressing the relationship between inflammation and IS complicated by AKI; second, although some prospective studies suggest a higher probability of AKI occurring in IS, they do not elucidate the causes and mechanisms of AKI in IS; third, there has been no more precise analysis based on the severity of the patient’s condition. Therefore, there is a need to identify clinical markers that can directly predict the occurrence of AKI in IS and have more specific accuracy.

Currently, no studies have indicated the relationship between NPAR and AKI in critically ill patients with IS. Therefore, this study investigates whether there is an association between NPAR and AKI in critically ill patients with IS.

## 2 Materials and methods

### 2.1 Data preprocessing

This study employed a cross-sectional design, utilizing the EICU Clinical Research Database (EICU-CRD) (version 2.0). The EICU Collaborative Research Database (EICU-CRD) is a large public database created in collaboration between Philips and the Massachusetts Institute of Technology (MIT) Laboratory for Computational Physiology (LCP). It encompasses routine data from over 200,000 patients admitted to various intensive care units across the continental United States in 2014 and 2015, collecting a wealth of high-quality clinical information, such as vital signs, nursing plan documents, disease severity, diagnostic information, and treatment information (12). The free availability of the data supports the development of many machine learning algorithms, decision support tools, and clinical research applications. The data used in this study from the EICU Collaborative Research Database is publicly available after registration, and its free availability also supports many applications, including the development of machine learning algorithms, decision support tools, and clinical research.

### 2.2 Definition of acute kidney injury in severe ischemic stroke patients during hospitalization

According to KDIGO (Kidney Disease: Improving Global Outcomes) standards:

1. Increase in serum creatinine ≥0.3 mg/dL (26.5 μmol/L) within 48 h.
2. Increase in serum creatinine to ≥1.5 times baseline within 7 days.
3. Decreased urine output, i.e., urine output <0.5 mL/(kg·h) within 6 h.

If any of the above conditions occur in ischemic stroke patients during hospitalization, they may be diagnosed with acute kidney injury. Additionally, there are other definitions or adjustments that may be made in specific studies based on actual conditions, but the KDIGO standard is one of the more widely used definitions for acute kidney injury.

### 2.3 Calculation method of NPAR

The following formula is used to calculate NPAR: NPAR = percentage of polymorphonuclear neutrophils ÷ albumin.

### 2.4 Exclusion inclusion criteria

**Figure 1:**
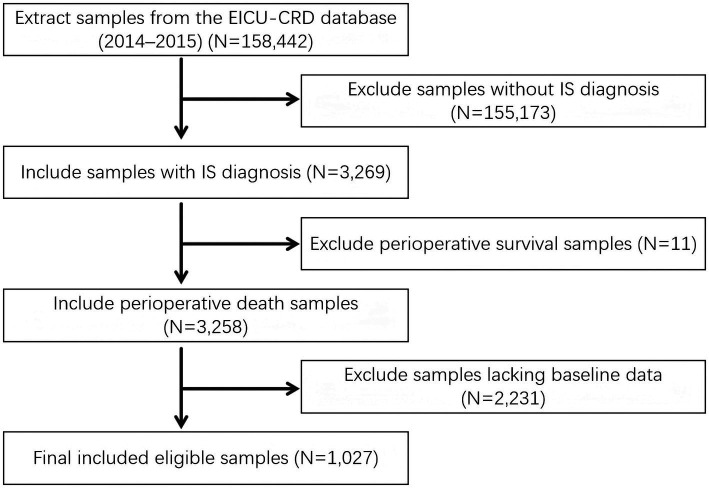
Flow chart.

Ischemic stroke (IS) was identified using ICD-9 codes 4,660, 34,661, 34,662, 34,663, 43,301, 43,311, 43,321, 43,331, 43,381, 43,391, 43,401, 43,411, 43,491, and ICD-10 code I63. Among the initially extracted 158,442 patients, we excluded the following: (1) non-IS diagnostic samples (*N* = 155,173), (2) samples lacking baseline data (*N* = 2,231), (3) samples of patients who survived during hospitalization (*N* = 11). The final study included 1,027 patients, among which 137 were severe IS patients with AKI during hospitalization, and 890 were severe IS patients who died without AKI (Figure 1).

### 2.5 Covariates

Based on previous literature, potential confounding factors were analyzed, including age, gender, height, race, weight, history of renal insufficiency, medication use (furosemide, iohexol, bumetanide), alanine aminotransferase, anion gap, aspartate aminotransferase, bedside blood glucose, bicarbonate, blood urea nitrogen, calcium, creatinine, eosinophil count, hematocrit, hemoglobin, lymphocytes, mean corpuscular hemoglobin concentration, platelet count, potassium, red blood cell count, red blood cell distribution width, total bilirubin, total protein, white blood cell count, heart rate, non-invasive diastolic blood pressure, sodium, body temperature, average non-invasive blood pressure, systolic blood pressure, and respiratory rate.

Age, sex, height, race, and weight were determined from the demographic variable file (DEMO) in the EICU-CRD survey. Ethnic groups were categorized as African American, Asian, Caucasian, Hispanic, and Native American. Sex was categorized as male and female.

### 2.6 Weighted logistic regression analysis

Weighted logistic regression was used to analyze the potential association between NPAR and AKI in severe IS patients during hospitalization. NPAR was included in the regression model as both continuous and categorical variables. This helps calculate odds ratios (OR) and their corresponding 95% confidence intervals (95% CI). NPAR was introduced into the model as a continuous variable. The original NPAR was divided into quartiles based on interquartile ranges, arranged from low to high. These quartiles were labeled as the first quartile (Q1), second quartile (Q2), third quartile (Q3), and fourth quartile (Q4). Further evaluation was conducted using these quartiles as categorical variables. When NPAR was treated as a categorical variable, the lowest quartile (Q1) was used as the reference. The study established three different statistical analysis groups: Model 1 represented the unadjusted model, while model 2 included adjustments for age, sex, height, race, weight, history of renal insufficiency, and furosemide use. Based on the adjustments in Model 2, Model 3 further adjusted for alanine aminotransferase, anion gap, aspartate aminotransferase, bedside blood glucose, bicarbonate, blood urea nitrogen, calcium, creatinine, eosinophil count, hematocrit, hemoglobin, lymphocytes, mean corpuscular hemoglobin concentration, platelet count, potassium, red blood cell count, red cell distribution width, total bilirubin, total protein, white blood cell count, heart rate, non-invasive blood pressure diastolic, and respiratory rate. All regressions included survey weights, and non-normally distributed continuous covariates were transformed using weighted quartiles. Additionally, interaction analyses were conducted to assess potential interactions between each subgroup and NPAR. *p*-values were adjusted for false discovery rate (FDR).

### 2.7 Clinical subgroup analysis

Subgroup analysis assessed whether the correlation between NPAR and AKI in severe IS patients during hospitalization differed across various subgroups. In this study, subgroup analysis and interaction effect analysis evaluated whether there were differences in associations between different sexes and ages. Forest plots were used to visually compare effect sizes and confidence intervals (CI) across multiple study results, providing a clearer reflection of the differences in OR (odds ratio) between the two groups and their 95% CI, helping us better understand the consistency and differences between different studies.

### 2.8 Restricted cubic spline (RCS)

Restricted cubic spline is a concept in statistics, particularly frequently used in regression analysis and curve fitting. It is a method for fitting and modeling continuous variables by dividing the data range into several intervals and using a cubic polynomial for fitting within each interval, thereby creating a smooth curve. These polynomials are smoothly connected across adjacent intervals, often with additional smoothness constraints to avoid sharp fluctuations in the curve. In statistical modeling, restricted cubic splines are commonly used to model the relationship between continuous variables and dependent variables. Especially in regression analysis, they allow capturing nonlinear relationships while maintaining smoothness and avoiding overfitting.

### 2.9 Statistical analysis

All data processing and analysis were completed using R software (version 4.4.2). Non-normally distributed continuous variables were described using median and interquartile range (IQR) to analyze baseline characteristics. Categorical variables were reported as sample counts and weighted percentages. To examine the variation in variable characteristics between NPAR groups (quartiles), the Wilcoxon rank-sum test was used for continuous variables, and the Rao-Scott chi-square test was used for categorical variables’ weighted percentages, providing a comprehensive description of the overall population. Statistical analysis was two-tailed, with *p* < 0.05 considered statistically significant.

## 3 Results

### 3.1 Baseline characteristics of the sample

A total of 1,027 samples were included, comparing the differences in baseline characteristics such as age, sex, height, race, and weight between IS patients who did not experience AKI during hospitalization and those who did. There was no difference between age, sex, height, race, weight, history of renal insufficiency, use of iohexol, and use of bumetanide during hospitalization of IS severe patients with or without AKI (*p*-value> 0.05). Other baseline data showed differences between the AKI group and the non-AKI group among IS patients during hospitalization, such as alanine aminotransferase (*p*-value<0.001), aspartate aminotransferase (*p*-value<0.001), bicarbonate (*p*-value<0.001), and white blood cell count (*p*-value<0.001), as shown in Table 1.

**Table 1:** Baseline characteristics of the study participants.

Variable	Overall	AKI	non_AKI	*p*-value^2^
	*N* = 1,027^1^	*N* = 137^1^	*N* = 890^1^	
Age	68 (14)	69 (13)	68 (14)	0.96
Gender				0.26
Female	496 (48%)	60 (44%)	436 (49%)	
Male	531 (52%)	77 (56%)	454 (51%)	
Height	169 (12)	170 (14)	169 (12)	0.14
Race				0.14
African American	125 (12%)	26 (19%)	99 (11%)	
Asian	21 (2.0%)	2 (1.5%)	19 (2.1%)	
Caucasian	827 (81%)	102 (74%)	725 (81%)	
Hispanic	52 (5.1%)	7 (5.1%)	45 (5.1%)	
Native American	2 (0.2%)	0 (0%)	2 (0.2%)	
Renalinsufficiency				0.19
Yes	170 (17%)	28 (20%)	142 (16%)	
No	857 (83%)	109 (80%)	748 (84%)	
Bumetanide				0.24
Drug - using group	104 (10%)	10 (7.3%)	94 (11%)	
Non - drug group	923 (90%)	127 (93%)	796 (89%)	
Furosemide				<0.001
Drug - using group	161 (16%)	39 (28%)	122 (14%)	
Non - drug group	866 (84%)	98 (72%)	768 (86%)	
Iohexol				0.4
Drug - using group	103 (10%)	11 (8.0%)	92 (10%)	
Non - drug group	924 (90%)	126 (92%)	798 (90%)	
NPAR	25 (8)	29 (10)	24 (7)	<0.001
weight	82 (23)	85 (25)	82 (23)	0.11
labalt	63 (267)	171 (499)	46 (205)	<0.001
labaniongap	11.1 (4.3)	12.1 (4.7)	11.0 (4.3)	0.001
labast	71 (304)	205 (647)	51 (198)	<0.001
labbedglucose	149 (72)	166 (89)	147 (69)	0.006
labbicarbonate	24.3 (3.9)	22.8 (5.0)	24.5 (3.6)	<0.001
labbun	23 (17)	39 (24)	20 (14)	<0.001
labcalcium	8.58 (0.74)	8.33 (0.91)	8.62 (0.70)	<0.001
labcreatinine	1.37 (1.52)	2.13 (1.92)	1.25 (1.41)	<0.001
labeos	1.27 (1.72)	1.10 (2.05)	1.30 (1.66)	0.002
labhct	37 (6)	35 (7)	37 (6)	0.01
labhgb	12.18 (2.31)	11.60 (2.52)	12.27 (2.26)	0.001
lablymphocytes	15 (10)	12 (12)	16 (10)	<0.001
labmchc	33.17 (1.34)	32.87 (1.47)	33.22 (1.32)	0.002
labplateletcount	213 (79)	191 (81)	217 (79)	<0.001
labpotassium	3.98 (0.61)	4.13 (0.73)	3.96 (0.59)	0.002
labrbc	4.07 (0.75)	3.93 (0.85)	4.10 (0.74)	0.013
labrdw	14.58 (2.03)	15.16 (2.16)	14.49 (1.99)	<0.001
labtotalbilirubin	0.77 (0.65)	0.92 (0.78)	0.74 (0.62)	0.027
labtotalprotein	6.27 (0.85)	5.92 (1.01)	6.33 (0.81)	<0.001
labwbc	11.1 (5.4)	12.7 (6.1)	10.9 (5.2)	<0.001
heartrate	85 (20)	89 (21)	84 (20)	0.004
nibp_diastolic	76 (19)	72 (18)	76 (19)	0.006
respiratoryrate	19.5 (5.8)	20.6 (6.5)	19.3 (5.7)	0.025
NPAR	25 (8)	29 (10)	24 (7)	<0.001

^1^Mean (sd) or Frequency (%).

^2^Wilcoxon rank sum test; Pearson’s Chi-squared test; Fisher’s exact test.

### 3.2 Relationship between AKI in severe IS patients during hospitalization and NPAR

Table 2 shows the association between AKI in severe IS patients during hospitalization and NPAR using a weighted logistic regression model. The Crude Model represents the relationship between AKI and NPAR in severe IS patients without covariates. The results indicate that NPAR is a risk factor for AKI in severe IS patients during hospitalization (NPAR, OR: 1.065, 95% CI: 1.043–1.087, *p* < 0.001); when compared to Q1, the OR for Q2 is 1.593 (95% CI: 0.818–3.188, *p* < 0.1766); the OR for Q4 is 4.914 (95% CI: 2.776–9.22, *p* < 0.001), indicating that the risk of AKI in severe IS patients during hospitalization increases from the first to the fourth quartile. Model 2 investigates the relationship between AKI and NPAR in IS severe patients during hospitalization after incorporating age, gender, height, race, weight, history of renal insufficiency, and furosemide use as covariates. Model 3 further incorporates alanine aminotransferase (ALT), anion gap, aspartate aminotransferase (AST), bedside blood glucose, bicarbonate, blood urea nitrogen (BUN), calcium, creatinine, eosinophil count, hematocrit, hemoglobin, lymphocytes, mean corpuscular hemoglobin (MCH), platelet count, potassium, red blood cell count, red blood cell distribution width (RDW), total bilirubin, total protein, white blood cell count, heart rate, and non-invasive diastolic blood pressure with respiratory rate as covariates. The results show positive correlations between these variables and perioperative AKI in IS severe patients: Model 2 (OR: 1.071, 95% CI: 1.049–1) (*p* < 0.001) and Model 3 (OR: 1.041, 95% CI: 1.007–1) (*p* = 0.0162). After grouping according to the quartile spacing, it was also found in Model 2 and Model 3 that compared with Q1, Q4 (Model 2 OR: 5.529, 95% CI: 3.08–10.514, *p*-value <0.001; Model 3 OR: 3.598, 95% CI: 1.482–9.12, *p*-value = 0.0056) had the highest OR value in Q4 group, indicating that when NPAR was ≥28.27, the probability of perioperative AKI in IS severe patients was higher, as shown in Table 2.

**Table 2:** Univariate logistic regression analysis of the association between NPAR exposure and Ischemic stroke with AKI.

Characterisitic	Exposure cutoff	Case (%)	Model 1			Model 2			Model 3		
			OR	95% CI	*p*-value	OR	95% CI	*p*-value	OR	95% CI	*p*-value
NPAR											
NPAR continuous			1.065	(1.043, 1.087)	*p* < 0.001	1.071	(1.049, 1.094)	*p* < 0.001	1.041	(1.007, 1.076)	0.0162
NPAR quantile											
Q1 (low)	< 19.71	257 (25.02%)	Ref	Ref		Ref	Ref		Ref	Ref	
Q2	19.71- < 22.92	256 (24.93%)	1.593	(0.818, 3.188)	0.1766	1.566	(0.796, 3.164)	0.1996	1.585	(0.722, 3.577)	0.2569
Q3	22.92- < 28.27	257 (25.02%)	2.886	(1.579, 5.537)	9e-04	3.032	(1.637, 5.891)	6e-04	2.378	(1.053, 5.584)	0.041
Q4 (high)	≥ 28.27	257 (25.02%)	4.914	(2.776, 9.22)	*p* < 0.001	5.529	(3.08, 10.514)	*p* < 0.001	3.598	(1.482, 9.12)	0.0056
*p* for trend					*p* < 0.001			*p* < 0.001			0.0028

OR, odds ratio; CI, confidence intervals; NPAR:Neutrophil percentage to albumin ratio.

NPAR: Q1: NPAR< 19.71; Q2: 19.71 ≤ NPAR<22.92; Q3: 22.92≤ NPAR<28.27; Q4: NPAR≥ 28.27.

The model 1 was the crude model.

The model 2 was adjusted by Age, Gender, Height, Race, Weight, Renalinsufficiency, Furosemide.

The model 3 was adjusted by Age, Gender, Height, Race, Weight, Renalinsufficiency, Furosemide, Alt, Aniongap, Ast, Bedglucose, bicarbonate, Bun, Calcium, Creatinine, Eos, Hct, Hgb, Lymphocytes, Mchc, Platelet count, Potassium, Rbc, Rdw, Total Bilirubin, Total Protein, Wbc, Heartrate, Nibp_diastolic, Respiratoryrate.

### 3.3 Risk of AKI in severe IS patients during hospitalization and NPAR’S RCS curve

**Figure 2:**
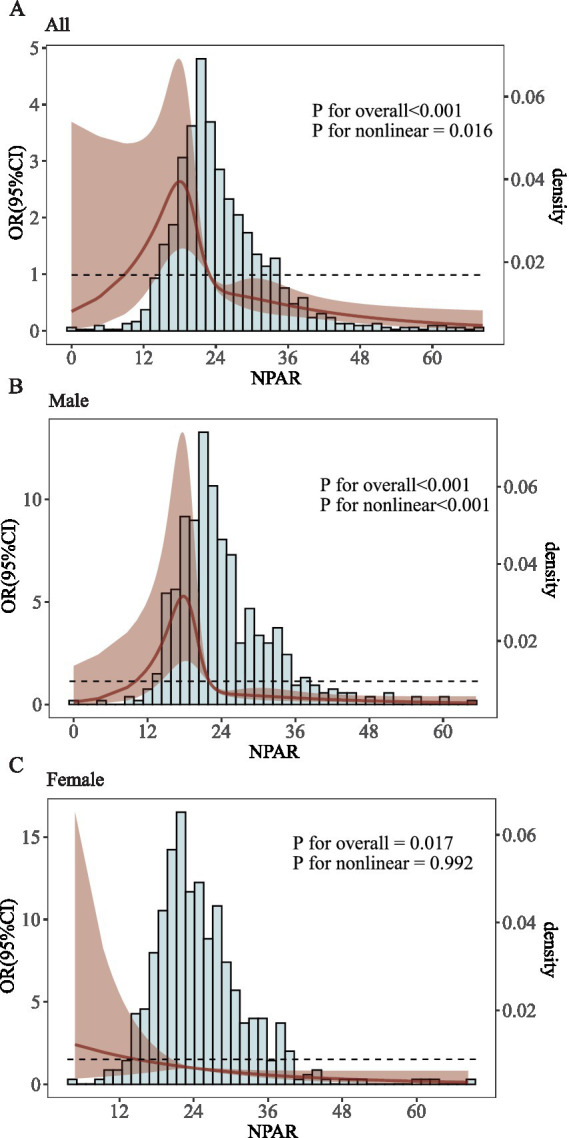
RCS curve of perioperative AKI and NPAR in IS patients. Subjects include male (Male adults) and female (Female adults). Covariates: Age, gender, height, race, weight, alanine aminotransferase (ALT), anion gap, aspartate aminotransferase (AST), bedside blood glucose, bicarbonate, blood urea nitrogen (BUN), calcium, creatinine, eosinophil count, hematocrit, hemoglobin, lymphocytes, mean corpuscular hemoglobin concentration, platelet count, potassium, red blood cell count, red blood cell distribution width (RDW), total bilirubin, total protein, white blood cell count, heart rate, non-invasive blood pressure (systolic/dipper) and respiratory rate.

The relationship between NPAR and the risk of AKI in severe IS patients during hospitalization was studied using a restricted cubic spline (RCS) curve, adjusting for all relevant covariates. There was a statistically significant correlation between NPAR and the risk of AKI in severe IS patients during hospitalization, exhibiting a nonlinear relationship (*p* for overall < 0.001, *p* for nonlinear = 0.016, Figure 2A). After subgroup analysis by sex, a significant correlation was found between NPAR and the risk of AKI in severe IS patients during hospitalization in males (*p* for overall < 0.001, *p* for nonlinear < 0.001, Figure 2B), while in females, a significant correlation was found but no nonlinear relationship was observed (*p* for overall = 0.017, *p* for nonlinear = 0.992, Figure 2C).

### 3.4 Relationship between NPAR and baseline characteristics by subgroup

**Figure 3:**
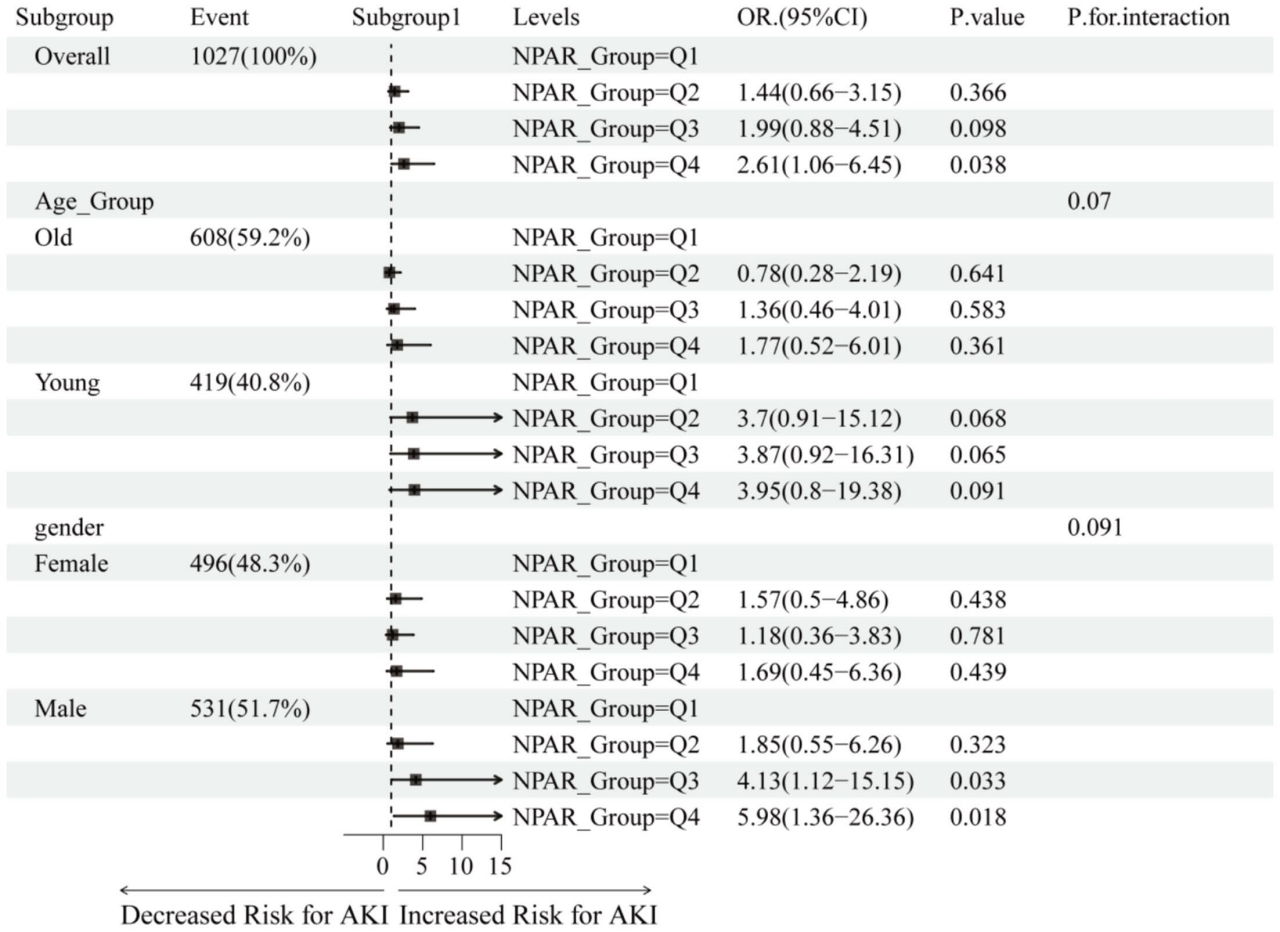
Relationship between baseline characteristics and NPAR and IS risk of AKI during hospitalization in severe patients. In the age subgroup, Young refers to age ≤65 years, and Old refers to age>65 years. Covariates include: age, gender, height, race, weight, alanine aminotransferase (ALT), anion gap, aspartate aminotransferase (AST), ambulatory blood glucose, bicarbonate, blood urea nitrogen (BUN), calcium, creatinine, eosinophil count, hematocrit, hemoglobin, lymphocytes, mean corpuscular hemoglobin concentration, platelet count, potassium, red blood cell count, red blood cell distribution width (RDW), total bilirubin, total protein, white blood cell count, heart rate, and non-invasive blood pressure (systolic/dipper) with respiratory rate.

Figure 3 to evaluate the relationship between NPAR and baseline characteristics across subgroups, subgroup analyses were conducted. The results showed no significant interactions were observed in any subgroup analysis (*p* > 0.05). Specifically, younger age groups demonstrated greater sensitivity to NPAR changes (OR: 8.03, 95% Cl: 2.19–29.42, *p*-value: 0.002), male patients showed higher responsiveness (OR: 3.35, 95% Cl: 1.07–10.48, *p*-value: 0.038), individuals without prior renal insufficiency history exhibited increased sensitivity (OR: 3.42, 95% Cl: 1.5–7.79, *p*-value: 0.003), and non-fluorsemide-treated patients exhibited heightened sensitivity (OR: 2.39, 95% Cl: 1.03–5.53, *p*-value: 0.042).

### 3.5 The predictive efficacy of NPAR for ischemic stroke combined with AKI was analyzed based on ROC curve

**Figure 4:**
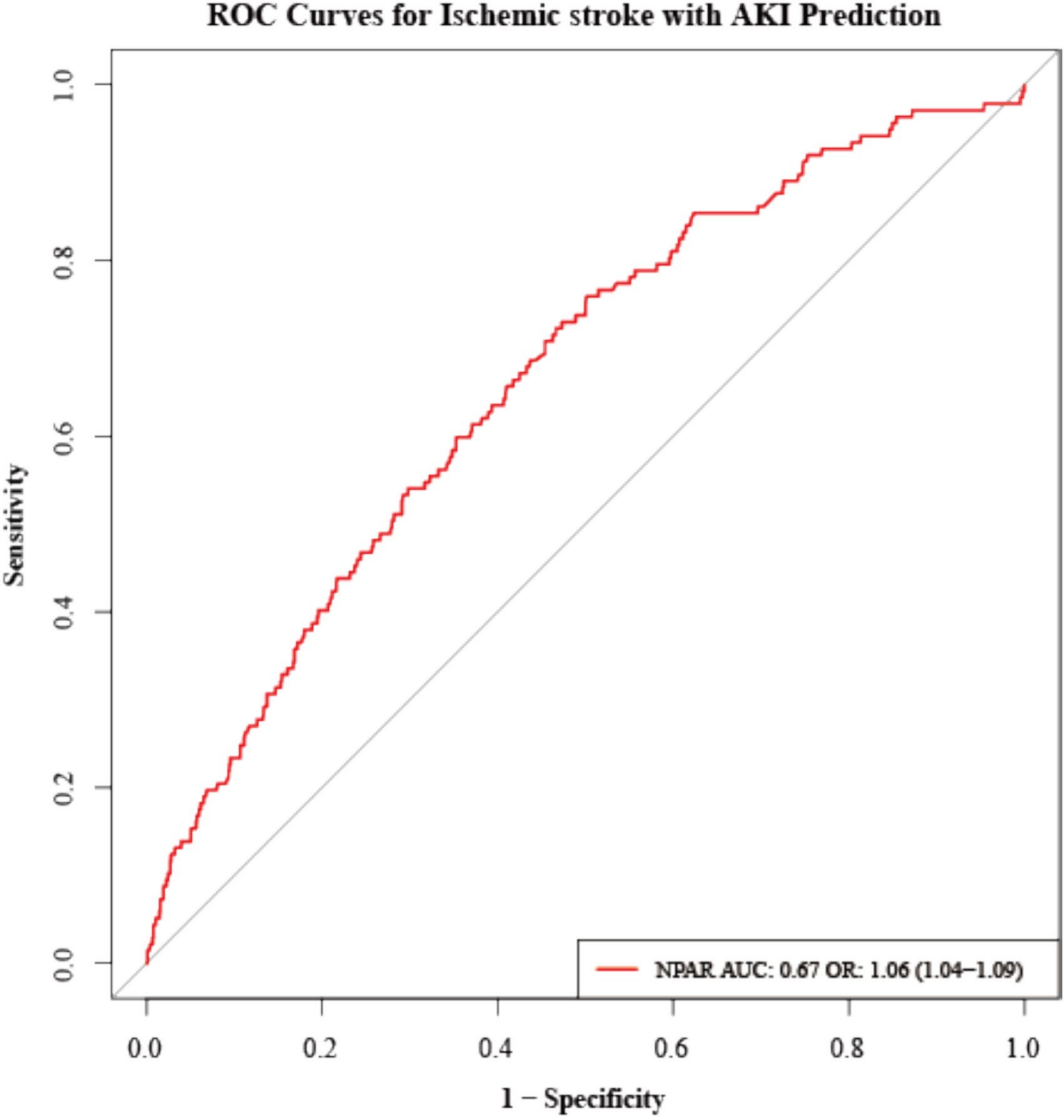
The ROC curve used to evaluate the predictive ability of NPAR for ischemic stroke combined with AKI.

In a predictive study of ischemic stroke complicated by acute kidney injury (AKI) (Figure 4), N-protein (NPAR) demonstrated clinical value. ROC curve analysis showed an area under the curve (AUC) of 0.666 with a 95% confidence interval of 0.62–0.71, indicating moderate predictive discrimination for AKI in ischemic stroke patients. The corresponding odds ratio (OR) was 1.064 (95% CI: 1.04–1.09), with an extremely low *p*-value (1.83 × 10^−9^), suggesting a significant correlation between elevated NPAR and increased AKI risk. The optimal cutoff value determined by the Jondal index was 24.87, achieving a sensitivity of 0.598 and specificity of 0.647 (Supplementary Table S1).

## 4 Discussion

The neutrophil percentage to albumin ratio (NPAR) is an indicator that comprehensively reflects the body’s inflammatory state and nutritional status. Neutrophils play a key role in the inflammatory response. When the neutrophil percentage increases, it usually indicates the presence of infection, inflammation, or tissue damage. Neutrophils participate in the clearance of pathogens and tissue repair by releasing reactive oxygen species, proteases, and cytokines. However, excessive activation of neutrophils may also lead to uncontrolled inflammatory responses, causing tissue damage and organ dysfunction. An increase in the neutrophil percentage in NPAR indicates a more active inflammatory response, which may be due to infection, autoimmune diseases, trauma, etc. Monitoring NPAR can provide timely insights into the patient’s inflammatory status, aiding clinical diagnosis and treatment (13).

Albumin is one of the main proteins in human plasma, synthesized by the liver. Plasma albumin concentration is used as an indicator of nutritional status and liver function. It plays important roles in maintaining plasma colloid osmotic pressure, transporting nutrients and drugs, and regulating acid–base balance. The level of albumin can reflect the body’s nutritional status and liver synthetic function. Low albumin levels are usually associated with malnutrition, chronic diseases, and liver dysfunction. In NPAR, the lower the albumin level, the higher the ratio, indicating that the patient’s nutritional status may be poor. Malnutrition can affect the patient’s immune function, wound healing, and recovery ability, increasing the risk of infection and complications (14).

Many studies have shown that NPAR is closely related to the prognosis of various diseases. In critically ill patients, high NPAR often indicates higher mortality and poor prognosis; additionally, elevated NPAR levels are significantly associated with increased all-cause and cardiovascular disease mortality risk in the general population (15). By dynamically monitoring changes in NPAR, treatment plans can be adjusted in a timely manner to improve patient prognosis. For example, for patients with high NPAR, proactive anti-infection treatment, nutritional support, and organ function protection measures can be taken to reduce inflammatory responses and improve albumin levels, thereby enhancing patient prognosis. NPAR reflects the interaction between inflammation and nutritional status in the body, integrating key components of immune response and nutritional health, making it a valuable tool for assessing disease severity and predicting outcomes across a range of clinical conditions. In patients with carcinoma of the colon, elevated NPAR values are associated with advanced disease stages, indicating its potential role as a predictive marker for prognosis and treatment response (16). NPAR has been identified as a significant factor in predicting outcomes for patients with diabetic foot infections and those undergoing major lower limb amputations, where high NPAR levels correlate with increased mortality risk (17). These findings underscore the importance of NPAR as a composite marker encompassing both inflammatory and nutritional dimensions, thereby providing a more comprehensive assessment of patient health status. In cases of sepsis, NPAR has been recognized as a significant marker correlated with the Sequential Organ Failure Assessment (SOFA) score, a widely used tool for evaluating sepsis severity, showing promise as a practical biomarker for early sepsis identification and management. This allows for timely intervention, thereby significantly improving patient survival rates (18, 19).

Recent studies have shown that a series of complex inflammatory responses occur in the body after stroke, including local and systemic inflammation. These responses not only affect the extent of brain tissue damage but are also closely related to the recovery process post-stroke (18, 19). Inflammation plays a dual role in IS. On one hand, the inflammatory response serves as the body’s natural defense mechanism against cerebral ischemia, capable of clearing damaged cells and promoting tissue repair. On the other hand, excessive inflammatory responses may lead to secondary damage, affecting patient prognosis. The inflammatory response plays a central role in the pathogenesis of AKI. Research indicates that during ischemia–reperfusion injury (I/R), renal tubular epithelium releases various pro-inflammatory factors, which not only exacerbate local inflammation but also lead to cell apoptosis and fibrosis (20). Additionally, damage to renal tubular cells triggers a series of alterations in cellular signaling pathways, including the activation of the NLRP3 inflammasome, further promoting renal inflammatory responses and injury (21). The mechanisms underlying IS combined with AKI are not yet fully understood, but existing studies suggest that ischemic injury, inflammatory responses, and oxidative stress play significant roles in this process (8). Following the onset of IS, the kidneys are affected by multiple factors, such as reduced renal blood flow, renal tubular injury, and renal interstitial inflammation, all of which collectively contribute to the occurrence of AKI. The systemic inflammatory response induced by cerebral ischemia can impair renal tubular function through various pathways, leading to renal tubular injury. Additionally, the overactivation of the sympathetic nervous system post-stroke may exacerbate kidney injury by increasing oxidative stress and apoptosis in renal cells (22). Furthermore, cerebral ischemia may aggravate kidney damage through the release of pro-inflammatory factors (23). Therefore, there exists a complex interplay between cerebral ischemia and kidney injury. Interventions targeting inflammatory responses may provide novel therapeutic strategies for improving renal function following cerebral ischemia.

Managing IS patients with AKI is more challenging, as the coexistence of both conditions can worsen the patient’s condition and increase the healthcare burden. AKI not only exacerbates the clinical manifestations of stroke but is also associated with higher mortality rates and poor functional prognosis (24). Studies have shown that the mortality rate of AKI patients within 30 days after a stroke is significantly higher than that of non-AKI patients. Various risk factors for AKI in IS patients have been identified, including advanced age, chronic kidney disease, diabetes, and hypertension (25); the severity of the stroke (NIHSS score) is positively correlated with the incidence of AKI, with more severe strokes leading to higher rates of AKI (26).

This study employed a cross-sectional research method to investigate routine data from over 200,000 patients admitted to various intensive care units across the continental United States from 2014 to 2015 in the EICU Clinical Research Database (EICU-CRD) (version 2.0). A sample of 1,027 patients was selected, including 137 severe ischemic stroke patients with acute kidney injury. The results indicate that an increase in NPAR is associated with an increased risk of acute kidney injury in severe ischemic stroke patients, with an odds ratio (OR) of 1.03, suggesting that NPAR can serve as an associated risk factor for acute kidney injury in severe ischemic stroke patients.

The occurrence of AKI in ischemic stroke patients is influenced by various risk factors. Many studies have shown that underlying renal dysfunction is a significant risk factor, with chronic kidney disease (CKD) patients being more prone to AKI after a stroke (24). Additionally, metabolic diseases such as hypertension, diabetes, and age are closely related to the occurrence of AKI (27). One study indicated that patients using diuretics had a significantly increased risk of AKI. Furthermore, the severity of the stroke (e.g., NIHSS score) is an important factor influencing the occurrence of AKI, with higher NIHSS scores correlating with a greater risk of AKI (28). Ischemic stroke not only affects brain tissue but also impacts kidney function through interactions in the brain-kidney circulation. Ischemia in the brain activates the sympathetic nervous system, leading to reduced renal blood flow and subsequent renal dysfunction. Research has shown that infarction in the right insular cortex is closely related to the occurrence of acute kidney injury, indicating that brain injury may affect kidney perfusion and function through neuroendocrine pathways (29). Additionally, inflammatory responses in the brain-kidney circulation play a crucial role; after ischemic stroke, inflammatory factors released from brain tissue can affect the kidneys through the bloodstream, leading to apoptosis and fibrosis of renal tubular cells (30). However, the predictive ability for complications in the aforementioned research results is insufficient; NIHSS is a scale used to analyze disease progression trends, which is relatively subjective, and the evaluation conditions for neuroendocrine factors are relatively high, potentially requiring costly examinations and increasing the number of invasive procedures for patients. NPAR is easily obtainable in clinical management and has been proposed as a novel indicator for assessing the risk and prognosis of IS; in the pathophysiological mechanisms of AKI, the infiltration of inflammatory cells and the release of inflammatory factors are important pathological processes (31). Therefore, this study focuses on the association between NPAR and acute kidney injury in severe ischemic stroke patients.

Neutrophils, as key components of the body’s immune response, play a central role in the inflammatory response of IS, and changes in their numbers can reflect the immune status of the body. Studies have shown that an increase in neutrophils is closely related to poor prognosis in IS patients (1). Meanwhile, albumin, as an important plasma protein, is associated with various pathological states, particularly in IS, where low levels of albumin are significantly associated with poor prognosis (32). Against this backdrop, the neutrophil percentage to albumin ratio (NPAR) has been proposed as a novel indicator for assessing the risk and prognosis of IS. Research has found that higher NPAR is associated with complications (such as infections) and poor functional prognosis after IS (33).

The clinical application of NPAR is not without limitations. A major challenge is the lack of standardized testing methods and universally accepted cutoff values, which may lead to discrepancies in results across different laboratories and *Homo sapiens* populations. Such inconsistency can compromise the reliability of NPAR as a clinical diagnostic tool (34). Furthermore, NPAR levels can be influenced by various non-disease factors, including age, baseline health status, and comorbidities, potentially distorting the interpretation of results (35). For instance, elderly patients or those with chronic conditions may naturally exhibit altered neutrophil counts or albumin levels, thereby complicating the assessment of their inflammatory status (36). Additionally, in specific *Homo sapiens* populations, such as patients with liver disease, careful consideration is required when interpreting NPAR due to potential confounding factors affecting neutrophil and albumin levels (37). Therefore, although NPAR shows promise as a biomarker, its application must be approached with caution, and further research is needed to establish clear guidelines for its use across diverse clinical settings.

This study also has certain limitations. The samples included were from a subset of cases admitted to various intensive care units in the continental United States in 2014 and 2015. Due to regional differences in medical standards and ethnic variations, the results of this study may not be representative of all ethnic groups globally. The data collected in this study were obtained from public databases, which did not explicitly specify the timing of neutrophil and albumin collection or whether dynamic monitoring was performed. Therefore, it is impossible to determine whether samples collected at different time points would affect the NPAR results, nor can the impact of fluctuating NPAR on the study outcomes be assessed. Regarding statistical analysis, although multivariate adjustments were conducted, certain clinical variables (such as baseline renal function and the use of nephrotoxic medications) could not be analyzed in this study due to incomplete data collection for these variables in the databases. The relationship between the severity of AKI (KDIGO staging criteria) and NPAR was not mentioned. Additionally, this study is retrospective, and with the rapid development of modern medicine, further prospective, multi-center, large-sample studies are needed to validate the conclusions of this study.

Research indicates that compared to single indicators, NPAR may possess stronger prognostic capability, making it a valuable tool for predicting outcomes across various diseases (38). This study suggests that an increase in NPAR raises the risk of AKI in severe IS patients and can serve as a clinical marker for predicting AKI in severe IS patients during clinical management.

## Data Availability

Publicly available datasets were analyzed in this study. The data can be found in the eICU Collaborative Research Database (eICU-CRD), https://eicu-crd.mit.edu/.
